# Peripheral Blood Mononuclear Cell Gene Expression Remains Broadly Altered Years after Successful Interferon-Based Hepatitis C Virus Treatment

**DOI:** 10.1155/2015/958231

**Published:** 2015-10-19

**Authors:** Paul Ravi Waldron, Mark Holodniy

**Affiliations:** ^1^VA Palo Alto Health Care System, Palo Alto, CA 94304, USA; ^2^Division of Infectious Diseases & Geographic Medicine, Stanford University, Stanford, CA 94305-5107, USA

## Abstract

*Background*. Inflammatory gene expression in peripheral blood mononuclear cells (PBMCs) is altered in chronic Hepatitis C Virus (HCV) infection. Duration of changes after pegylated interferon- (peg-IFN-) based HCV treatment is unclear. *Methods*. PBMC mRNA expression of 184 inflammatory response genes was analyzed (nCounter GX Human Inflammation Kit, Nanostring) from peg-IFN treatment nonresponders (NR, *n* = 18), sustained virologic responders (SVR, *n* = 22), and spontaneous clearers (SC, *n* = 15). Logistic regression was used for comparison. *Results*. Median time from last treatment was 2 and 2.7 years in SVR and NR, respectively (*p* = NS). Mean mRNA counts were significantly different for 42 and 29 genes comparing SVR to SC patients and NR to SC, respectively, and no genes comparing SVR to NR. Differential expression of 24 genes was significantly different in both SVR and NR groups compared to SC. Among these 24 acute and chronic inflammatory cascade genes, significant upregulation was noted for proinflammatory transcription regulators *Fos*, *CEBPB*, and *MyD88* in SVR and NR compared to SC. *HDAC4* was significantly downregulated in SVR and NR compared to the SC group. *Conclusions*. PBMC inflammatory gene expression patterns in SVR resemble NR more than SC patients. A generalized inflammatory response persists in PBMCs long after successful peg-IFN treatment for HCV infection.

## 1. Introduction

Hepatitis C Virus (HCV) infection affects over 185 million individuals worldwide and approximately 5.2 million in the US. HCV is a major cause of severe disease in the US, with mortality rates of 4.7 per 100,000 reported in 2010 [1–4]. Approximately 70% of people infected with HCV develop chronic infection and 30% are known to spontaneously clear the infection; however, these rates vary by ancestry, gender, and other factors [5–7]. Chronic infection with HCV results in hepatic inflammation, microfibrosis and may ultimately cause cirrhosis, hepatocellular carcinoma, and death due to liver failure.

Prior to December 2013, the standard of care for HCV consisted of 24–48 weeks of pegylated interferon (peg-IFN) and ribavirin (RBV) with a HCV protease inhibitor added for genotype 1 infection [8, 9]. After December 2013, interferon-free regimens have been approved with directly acting antivirals (DAAs) [10]. Treatment outcomes have been traditionally defined as either nonresponse (NR) (which includes null response and early relapse) or sustained virologic response (SVR) defined as a persistent undetectable serum HCV RNA or viral load 24 weeks after the conclusion of treatment [8, 9]. NR is considered a treatment failure, whereas SVR has been considered a functional cure and has been associated with improved clinical outcomes [11].

However, an estimated 3% of patients will relapse after achieving SVR with reemergence of the same virus after peg-IFN/RBV treatment [12]. It is not yet clear how new DAAs will affect this rate. Reasons for late relapse remain unclear. It is most likely that a viral reservoir remains in the liver [13]. Peripheral blood mononuclear cells (PBMCs) have been cited as a possible viral reservoir with previous studies suggesting that 9–26% of SVR patients may have residual virus in PBMCs [13–19]. However, HCV infection of PBMCs is controversial, with some studies demonstrating that there may not be true replicative infection within PBMCs and, rather, that the presence of viral RNA may be due circulating virions in the serum passively diffusing into cells [20, 21].

It has been demonstrated that genes involved in the inflammatory response are dysregulated in the setting of chronic HCV infection and liver disease with resulting differences in circulating cytokine profiles [22, 23]. However, it is an open question how durable these changes are after interferon treatment. A prior study by our group demonstrated that patients with SVR had significantly different serum cytokine profiles compared to patients exposed to HCV but with no evidence of ever having clinical infection (spontaneous clearance, SC) [22]. This data suggested continued activation of both innate and adaptive antiviral response mechanisms [22]. Prior studies have examined gene expression of a limited number of genes in PBMCs using PCR-based assays in SVR patients [24, 25]. Newer technologies employ multiplex oligonucleotide based arrays to directly quantify and count copy numbers in a sample without the variation inherent in techniques that require nucleic acid amplification [26, 27].

We sought to determine if there was a persistent inflammatory response in patients who attained SVR after interferon treatment. To this end, we used a multiplex array to investigate PBMC gene expression in patients previously treated for HCV to determine if differences in mRNA transcription persisted despite successful treatment.

## 2. Materials and Methods

### 2.1. Ethics Statement

The study was approved by Stanford University institutional review board and was conducted under guidelines established by the Declaration of Helsinki. Written informed consent was obtained from all patients.

### 2.2. Cohorts and Design

We performed a cross-sectional study of patients with a history of HCV infection from the Veterans Affairs Palo Alto Health Care System (VAPAHCS). Potential study participants were identified from a clinical case registry of previously HCV antibody tested and/or treated patients at the VAPAHCS and were recruited into the study from June 2010 to September 2014. Two arms of patients were recruited. The first arm included patients with HCV infection who had completed interferon-containing HCV treatment between 6 months and 5 years prior to study enrollment. These patients were categorized as either SVR or rebound/relapse/nonresponse (NR). SVR patients were defined as having an undetectable HCV viral load (Abbott RealTime HCV, Abbott Molecular, Des Plaines, IL) at least 24 weeks after completing therapy. NR patients were defined as any patient who received HCV therapy and either never had an undetectable HCV viral load or had a recurrence of viremia either during or subsequent to treatment. Also included were VAPAHCS patients who were exposed to HCV and spontaneously cleared the virus (SC). SC patients were defined as antibody positive by commercial assay (ARCHITECT Anti-HCV assay, Abbott Molecular, Des Plaines, IL) but who had 2 undetectable HCV RNA viral load results at least 1 month apart without any treatment. Patients who had HIV coinfection, were on any systemic immunomodulatory medications (including glucocorticoid, anti-TNF-*α* antibodies, and antineoplastic agents), and could not attend study visits, or from whom sufficient human nucleic acid could not be isolated for analysis, were excluded.

### 2.3. Descriptive and Clinical Patient Information

Patient race/ethnicity was based on self-report. Both medical records and patient interview were used to obtain additional information on age, medication use, and concurrent medical conditions. To approximate the stage of liver disease, the fibrosis-4 (FIB-4) score as described by Vallet-Pichard et al. was calculated using each patient's age, aspartate aminotransferase (AST) and Alanine transaminase (ALT) serum levels, and platelet count. Any patient with a FIB-4 score greater than 3.25 was considered to have significant fibrosis comparable to a FibroTest score of F3-F4 [28]. An age adjusted Charlson Comorbidity Index score was also calculated for each person [29]. For the purposes of the Charlson score, all patients with prior HCV, except for those with a FIB-4 score greater than 3.25, were identified as having mild liver disease based on the fact that they were all chronically infected with HCV. Those with a FIB-4 score greater than 3.25 were identified as having moderate to severe liver disease. Other conditions identified in one or more patients included diabetes, chronic obstructive pulmonary disease, connective tissue disease, peripheral vascular disease, lymphoma, any tumor, myocardial infarction, and congestive heart failure. Data on statin and systemic prescription nonsteroidal anti-inflammatory drugs (NSAID) use at the time of the blood draw was collected and tabulated. Over-the-counter NSAID use could not accurately be accounted for in all cases, so it was not included.

### 2.4. Sample Preparation

All patients included in the analysis had at least one blood draw at the time of enrollment. A smaller subset of patients in the SVR and NR groups underwent a second blood draw 3–6 weeks later as an internal control to ensure that study measurements were stable over time. PBMCs were isolated from blood collected in Cell Preparation Tubes (CPT) (Becton, Dickinson and Company, Franklin Lakes, NJ) per manufacturer instructions and stored at −80°C. Total RNA was extracted from 2.0 × 10^6^–5 × 10^6^ cells using AllPrep DNA/RNA Mini Kits (Qiagen, Valencia, CA) as per manufacturer instructions and placed into 50 *μ*L of RNase-free water. Concentration of total RNA was measured by spectrophotometry and then concentrated or diluted to a target concentration of between 20 and 60 ng/*μ*L.

### 2.5. Measurement of Gene Expression via Microarray

Gene expression was directly measured via counts of corresponding messenger RNA (mRNA) in each sample using an nCounter (Nanostring, Seattle, WA) GX human inflammation kit, which is a multiplex assay for 184 genes involved in the human inflammatory response (listed in supplemental Table 1 in Supplementary Material available online at http://dx.doi.org/10.1155/2015/958231). The nCounter system allows for direct detection and counting of nucleic acid via reporter probes appended with multiple fluorophore barcodes and biotinylated capture-probes that attach to microscopic beads, which are then affixed to lanes in a translucent cartridge and read in an optical scanner [26]. Batches of 12 separate samples at one time were prepared as per manufacturer instructions, with 100–300 ng of total RNA hybridized with probes at 65°C for 16–18 hours before being placed into the automated nCounter Prep Station (Nanostring) in which samples were affixed to cartridges. Cartridges were then immediately placed into the nCounter Digital Analyzer (Nanostring) optical scanner and read at a goal resolution of 550 Fields of View (FOV), which is the maximum resolution for this instrument.

### 2.6. Statistical Analysis

The raw Nanostring gene expression data were normalized using negative controls, positive controls, and housekeeping genes via nSolver version 2.0 software (Nanostring). The arithmetic mean plus 2 standard deviations (SDs) of the internal negative controls in each sample was subtracted from the gene expression count to ensure that any nonspecific mRNA detection was excluded. Values below zero were included as zero for the analysis. The geometric mean of the 6 internal positive controls was used to normalize the data so that comparisons could be made across samples and to minimize distortion from batch effects. Gene expressions were then normalized using the geometric mean of 5 housekeeping genes (GAPDH, CLTC, HPRT1, PGK1, and TUBB) chosen due to their consistent expression in PBMC. Batch effects were assessed with a confirmatory mixed batch of samples selected from each prior run with adjustments made as appropriate. The in-group means of gene expression count were compared via two-tailed pairwise analysis with nonparametric distribution assumed. Given the number of simultaneous tests and an expected increase in type 1 error, a Bonferroni corrected threshold *p* value of <0.001 was used to indicate significance. A Spearman correlation was used to cluster samples comparing overall expression levels. Logistic regression was used to compare the distribution of mean expression across groups. Age, FIB-4, age adjusted Charlson Index scores, and time since treatment were compared between groups with Kruskal-Wallis test for nonparametric data. Chi-squared tests were performed for comparison of categorical data such as sex, race, and HCV genotype.

## 3. Results

### 3.1. Study Participants

In the final analysis, 55 patients (18 NR, 22 SVR, and 15 SC patients) met both inclusion and exclusion criteria and were included in the study. After an initial database search and contact by mail, 79 patients were initially screened by phone for inclusion in the study. Of those, 3 patients were excluded due to refusal to participate, 5 patients were excluded due to receipt of interferon prior to the study period (before 12/2008), 8 were unable to participate in their initial visit, 1 patient was consented but was unable to complete blood draw, 1 patient was consented but RNA was not recovered from his samples, 3 patients were excluded due to the presence of immunomodulatory medications, and 3 patients were excluded due to the presence of HIV. Demographic and clinically relevant data including HCV genotype, FIB-4 and age adjusted Charlson Comorbidity scores, and statin or NSAID use are reported in Table 1. The SC population was significantly younger than the other two groups, with a median age of 58 years compared to 62 and 63 years (*p* = 0.047). Due to VAPAHCS patient demographics, whites and males were overrepresented in this study when compared to the overall US HCV population, although there were no significant differences between groups. As expected, FIB-4 scores were significantly higher in the NR group compared to SC or SVR (*p* = 0.001). The FIB-4 score was similar between SVR and SC patients. Age adjusted Charlson Comorbidity scores were significantly different between the three groups (*p* < 0.001); however, the difference appeared to be driven primarily by the amount of nonsevere liver disease (1 point) in the SVR group and cirrhotic liver disease in the NR group (3 points). Rates of prescription statin and NSAID use were similar between groups (*p* = 0.761 and 0.948, resp.). Between the SVR and NR group, there was a significantly higher percentage of genotype 1 patients in the SVR group (*p* = 0.027), which was unexpected and likely due to local treatment patterns in the last 5 years at VAPAHCS after the introduction of HCV protease inhibitors in 2011. Time from last interferon treatment (median 2.0 years in SVR group, 2.6 years in NR, *p* = 0.348) and type of treatment (*p* = 0.116) were not significantly different between groups.

### 3.2. Gene Expression

As many inflammatory mediators are not primarily transcribed in PBMC, not all genes assessed had significant transcription as detected by our primary assay. Of 184 inflammatory mediator genes assessed, 127 had significant expression levels (mean copy numbers >10 in any group) for reliable detection after normalization with negative controls, positive controls, and housekeeping genes and were included in further analysis. This null expression group included important inflammatory mediators primarily expressed in neutrophils, endothelial cells, tissue based macrophages, or other tissues, including IL-1A, IL-4, IL-6, and interferons *α* and *β*. A full list of assessed genes and expression is available in supplemental Table 1.

### 3.3. Clustering and Logistic Regression

**Figure 1:**
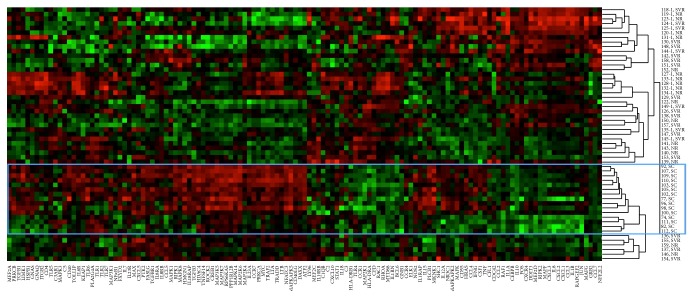
Gene expression heat map with sample clustering based on Spearman correlation (spontaneous clearance cluster highlighted).

When analyzed as individual samples, it was observed that overall pattern of gene expression between SVR and NR patients was highly similar, while SC patients differed from the other two groups. A heat map of gene expression with clustering using a Spearman correlation is shown in Figure 1 and is notable for the uniform clustering of SC patients together.

**Figure 2:**
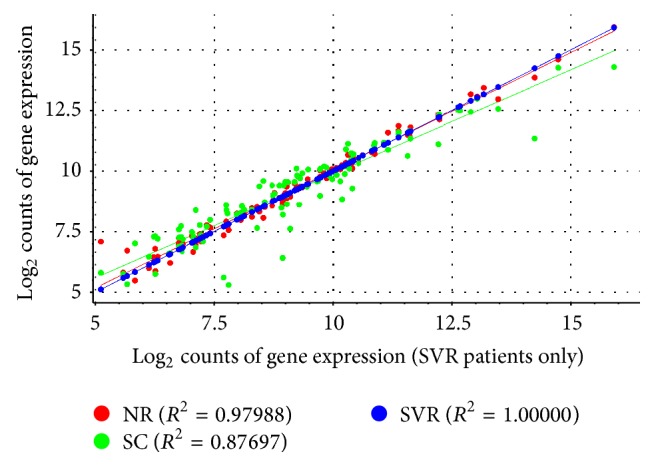
Scatter plot of mean counts with regression.

A scatter plot using logistic regression is shown in Figure 2. It is of note that the best-fit line for mean expression of SVR genes maps precisely onto that of NR genes with an *R* ^2^ of 0.98, while the correlation between SVR and SC gene expression was reduced with an *R* ^2^ of 0.88.

### 3.4. Comparison of Individual Gene Expression between Groups

Using a significance threshold of <0.001 (corrected for multiple testing), mean mRNA counts were significantly different for 42 genes when comparing SVR to SC patients, 29 genes comparing NR to SC, and no genes comparing SVR to NR. Differential expression of 24 genes was significantly different in both SVR and NR groups when compared to SC and is shown in Tables 2 and 3. Expression of 12 genes was upregulated and expression of 12 genes was downregulated. A full data set is available in supplementary Table 2.

Specific expression patterns were suggestive of increased transcription factor activation for promoters of cellular proliferation in PBMCs and resulting innate immune activity in NR and SVR patients versus SC patients. Although all 24 genes that were differentially expressed in the same direction in NR and SVR patients versus SC patients are potentially important inflammatory cascade regulators and effectors, a few of these are worth special mention as they may be of particular importance in interferon mediated immune response. It is of note that AP-1 components* Fos* (approximately 3-fold higher in both SVR and NR versus SC, *p* < 0.001 for both) and* Jun* (2.2-fold and 2.4-fold in both SVR and NR versus SC, *p* = 0.054 and 0.028, resp.) were both elevated as was* CEBPB* (*CCAAT/enhancer-binding protein beta*) (1.9-fold and 1.8-fold in both SVR and NR versus SC, resp., *p* < 0.001 for both). AP-1 is a known transcription factor for Toll-like receptor 4 (*TLR4*), which had a mild but nonsignificant elevation of transcription in the SVR and NR groups compared to SC (fold changes of 1.2 and 1.2, *p* = 0.04 and 0.02, resp.). Significant elevations in transcription of* TLR4* downstream effector Myeloid differentiation primary response gene 88 (*MyD88*) were also seen (fold changes of 1.2 and 1.6, resp., *p* < 0.001 for both) [30, 31]. The anti-inflammatory cytokine gene* IL-10* was not highly expressed in any group (counts < 25 in all groups, no transcription in SC). However, there was a significant elevation in both SVR and NR versus SC, perhaps consistent with feedback regulation from stimulation of the* MyD88* pathway [32]. It is of note that some transcription factors that favor an adaptive response including Histone Deacetylase 4 (*HDAC4*) were downregulated in SVR and NR compared to the SC group (*HDAC4* fold changes of 1.4 and 1.3, resp., *p* < 0.001 for both) [33].

## 4. Discussion

We found that the pattern of PBMC gene expression in SVR patients, who received peg-IFN treatment a median of 2 years prior, more closely resembles NR patients with active HCV infection than SC patients who have been exposed to HCV but are uninfected and who have no history of chronic viral infection or prolonged peg-IFN exposure. Given that there is ongoing viremia in NR patients and SVR patients have achieved clinical cure, it may be expected that NR patients would have a different pattern of expression in PBMCs from SVR patients; however, we found no difference. This indicates that there is a durable inflammatory transcriptional response in PBMCs that persists long after treatment. The specific pattern of gene expression demonstrates that genes related to innate immune activation such as* Fos*,* CEBPB*, and* MyD88* are more transcriptionally active in both SVR and chronically viremic patients, while adaptive immune mediators such as* HDAC4* are downregulated [30–33]. The pattern of activation found in this data can be understood in the light of the findings of Dill et al. who demonstrated in liver tissue that although the JAK/STAT pathway is transiently activated in response to pegylated IFN-*α*, other secondary pathways soon become the main mediators of effector response after sustained therapy and maybe responsible for the benefit of the pegylated form of the molecule [34].

A potential explanation for this pattern of activation may be liver disease itself and prior injury from chronic HCV infection. Lack of relative improvement in fibrosis after treatment may be an important predictor of outcomes in SVR patients [35]. Gene expression of macrophages from liver tissue has demonstrated upregulation of chemotactic factors and proinflammatory cytokines such as IL-8 in prior studies [36]. However, in this study population, the fibrosis scores of SVR patients were similar to those of SC patients and significantly different from chronically HCV infected NR patients, indicating that continued hepatic injury is most likely not occurring. This is also consistent with what is known about liver disease outcomes in SVR patients as 5-year mortality and progression to cirrhosis drop significantly. Pairing peripheral PBMC gene expression data with concurrent gene expression from liver macrophages along with histology from liver biopsy samples may be a useful line of further investigation.

Another possible explanation is a durable effect from long-term exogenous peg-IFN exposure. There is no significant body of prior literature suggesting this. However, HCV infected patients who achieve SVR receive much longer exposure to exogenous peg-IFN therapy than any other patient group, and this pattern of activation may mimic chronic viral infection, particularly when compared to NR group that also had an extended duration (although significantly less than the SVR group) of exogenous peg-IFN. The addition of a treatment naive chronically HCV infected comparison group may be useful in future studies to explore this possibility. A similar study in patients who receive interferon-free treatment regimens and obtain SVR may also resolve this hypothesis.

The explanation for this data with the most salient clinical consequences is either intra- or extrahepatic reservoirs of residual HCV infection. As demonstrated by Hara et al., late relapse in many cases is likely due to reemergence of prior infection rather than reinfection with a different strain [12]. As noted above, several studies have investigated the possibility of persistent viral reservoirs, including PBMCs themselves, although true infection of PBMC is controversial [13–21].

Two previous studies have used RT-PCR to assay PBMC gene expression in SVR patients and postulated residual infection as the etiology. Pham et al. [24] assessed transcripts of 9 cytokines in 22 chronically HCV infected patients and 29 patients with resolved disease compared with 15 healthy controls and found significant elevations in* IFN-α* and* TNF-α* in “resolved patients” versus chronically infected patients. However, the “resolved group” included both SVR and SC patients grouped together, and the chronic infection group included both treatment experienced and treatment naive patients [24]. Our study found negligible expression of* IFN-α* transcripts and no significant difference between groups in* TNF-α*. Radkowski et al. analyzed 25 cytokine transcripts in 49 patients with SVR divided into a group with residual infection as defined by very low levels of HCV in serum or PBMC detected with a home-brew ultrasensitive RT-PCR assay or by presence of NS3 protein via immunofluorescence in PBMC versus those without. There was a high rate (46.9%) of residual viremia found in this study, and in those patients there were significant elevations in* IL-6, IL-8, IL-12,TNF-α,* and* MIP-1β* (*CCL4*) when compared to SVR patients without evidence of residual virus [25]. Our study did not find significant differences between groups in the transcription of* IL-12*,* TNF-α*, or* CCL4* and did not detect significant transcription of* IL-6* in any group. We did find, however, that* IL-8* appeared upregulated in both SVR and NR patients compared to SC with fold changes of 7.49 and 5.73 (*p* = 0.005 and 0.08, resp.), although these changes did not reach the threshold for significance in the final analysis.

Our study differs from prior work in several ways, using an entirely different methodology to assay gene expression of many more genes simultaneously as well as a fundamentally different way of defining comparison groups, which may explain differences in results. Ours is the only study to assay expression of upstream genes whose products regulate overall transcription patterns rather than only transcripts for effector cytokines, which are not always reliably expressed in PBMCs as opposed to other tissue compartments.

Our study had several important limitations. The demographics of our patient population are not representative of the US HCV infected population at large in terms of gender or race; therefore, generalizability may be limited. The small numbers in each comparison group may not have allowed sufficient power to show a difference and increased the likelihood of type II error. As with any multiplex detection system reliant on binding to probes, uniform annealing conditions across 184 probe sets with different sequences may lead to falsely higher counts in probe/target sets with higher affinity and falsely lower or null counts in probe/target sets with lower affinity. Another important limitation is that the time since infection for the SC patients is unknown. This data is not obtainable due to the fact that acute infection with HCV is most often asymptomatic and a positive HCV antibody with negative serum HCV RNA PCR was identified in our SC sample on routine screening for at risk populations after the acute phase of infection. There is also a large distribution (6 months to 5 years) in the time since treatment for both the NR and SVR patients; however, the median time since treatment is not significantly different between groups; therefore, we believe these groups are comparable. In addition, our study is limited by assaying the total blood PBMC population as opposed to specific mononuclear cell subpopulations or liver tissue where important changes may be taking place. However, we believe changes in PBMC mRNA expression are important for our understanding of the systemic immune response after HCV treatment.

Further investigation is warranted to explain why interferon treated patients who obtain SVR and a functional cure have a similar pattern of macrophage and lymphocyte gene expression compared to those with chronic HCV infection who did not respond to treatment and also had prior exogenous interferon exposure. This is an important question in the DAA era of HCV therapy, as retreatment is now possible for patients with late relapse, and further monitoring of peg-IFN treated patients with SVR may be useful to identify late relapse. This may also be clinically important for monitoring peg-IFN treated patients for long-term consequences of prior infection such as progression to cirrhosis and HCC as well as immunogenic consequences of HCV infection such as cryoglobulinemia and porphyria cutanea tarda. Further studies investigating the same phenomenon in patients treated with interferon-free regimens are warranted.

## 5. Conclusion

Patients who achieved clinical cure of HCV infection with peg-IFN based regimens appear to have PBMC gene expression patterns that closely resemble chronically HCV infected patients who have failed treatment and are markedly different from patients who spontaneously clear their HCV infection. It is therefore reasonable to conclude that an inflammatory response persists in PBMCs long after successful peg-IFN treatment for HCV infection. Further study is warranted to better understand why this is occurring.

## Supplementary Material

The supplementary material consists of a spreadsheet containing the normalized gene expression counts for each gene investigated for each individual sample. Included are the counts for the housekeeping genes along with positive and negative controls used in normalization. The rightmost columns represent the p-values for pairwise comparisons between groups of each gene and are color coded by level of significance.

## Figures and Tables

**Table 1:** Demographical and clinical characteristics of subjects.

	SC	SVR	NR	*p* value
	*N* = 15	*N* = 22	*N* = 18	
Age, median (range)	58 years (41–67)	63 years (47–67)	62 years (50–69)	0.047 (Kruskal-Wallis)
Gender, number (%)				
Male	14 (93%)	21 (95%)	18 (100%)	0.57 (Chi-squared test)
Female	1 (7%)	1 (5%)	0	
Race, number (%)				
White	11 (73%)	15 (68%)	13 (72%)	0.975 (Chi-squared test)
Black	2 (13%)	4 (18%)	2 (11%)	
Other or mixed	2 (13%)	3 (14%)	3 (17%)	
Fib 4, median (range)	1.34 (0.78–3.74)	1.51 (0.85–3.69)	3.395 (1.02–14.18)	0.001 (Kruskal-Wallis)
Age adjusted Charlson score, median (range)	2 (0–6)	3 (1–9)	4 (2–10)	<0.001 (Kruskal-Wallis)
Prescription NSAID use, number (%)	4 (27%)	5 (23%)	4 (22%)	0.948 (Chi-squared test)
Statin use, number (%)	3 (20%)	3 (14%)	2 (11%)	0.762 (Chi-squared test)
HCG genotype, number (%)				
1	NA	20 (91%)	9 (50%)	0.027 (Chi-squared test)
2	NA	2 (9%)	5 (28%)	
3	NA	0	3 (17%)	
4	NA	0	1 (5%)	
HCV treatment, type				
Ribavirin/peg-interferon	NA	10 (45%)	13 (72%)	0.116 (Fisher exact test)
Ribavirin/peg-interferon + protease inhibitor	NA	12 (55%)	5 (28%)	
Time since last interferon treatment, median (range)	NA	721.5 days (217–2264)	972 days (357–2219)	0.348 (Mann-Whitney)
Weeks of interferon, median (range)	NA	48.0	21.5	0.003 (Mann-Whitney)

NA: not available.

**Table 2:** Genes with significantly different (*p* < 0.001) upregulation in both SVR and NR when compared to SC.

Gene ID	SC mean count	SVR mean count	NR mean count	Fold change SVR/SC	Fold change NR/SC	Fold change SVR/NR	*p* value SC v SVR	*p* value SC v NR	*p* value SVR v NR
MAP2K1	938	1205	1197	1.3	1.3	1	9.88*E* − 08	1.57*E* − 07	0.793
FOS	19984	61310	62471	3	3.1	−1.0	1.68*E* − 07	7.17*E* − 07	0.834
CEBPB	1571	3033	2873	1.9	1.8	1.1	4.12*E* − 07	9.60*E* − 06	0.608
RAC1	809	1006	1083	1.2	1.3	−1.1	1.13*E* − 05	4.74*E* − 04	0.200
HLA-DRA	19648	27320	24871	1.4	1.3	1.1	1.78*E* − 05	6.44*E* − 04	0.085
MYD88	1868	2288	3060	1.2	1.6	−1.3	2.42*E* − 05	2.40*E* − 05	0.0017
GNB1	5733	6570	6537	1.2	1.1	1.0	3.35*E* − 05	3.67*E* − 04	0.912
RHOA	2575	3159	3562	1.2	1.4	−1.1	7.97*E* − 05	1.70*E* − 05	0.048
CFD	199	338	364	1.7	1.8	−1.1	8.91*E* − 05	4.70*E* − 05	0.474
IL10	1	20	13	12.9	8.2	1.6	1.08*E* − 05	9.93*E* − 04	0.110
CCR1	503	845	1048	1.7	2.1	−1.2	1.22*E* − 05	4.59*E* − 05	0.109
IL1RN	389	625	852	1.6	2.19	−1.36	9.08*E* − 04	1.40*E* − 04	0.058

**Table 3:** Genes with significantly different (*p* < 0.001) downregulation in both SVR and NR when compared to SC.

Gene ID	SC mean count	SVR mean count	NR mean count	Fold change SVR/SC	Fold change NR/SC	Fold change SVR/NR	*p* value SC v SVR	*p* value SC v NR	*p* value SVR v NR
MAPK14	1896	1225	1292	−1.6	−1.5	−1.1	2.00*E* − 09	5.28*E* − 08	0.273
MAP3K7	899	606	602	−1.5	−1.5	1.0	3.58*E* − 08	5.98*E* − 08	0.943
RPS6KA5	660	349	319	−1.9	−2.1	1.1	1.26*E* − 07	2.57*E* − 08	0.320
CREB1	1070	711	701	−1.5	−1.5	1.0	7.08*E* − 07	4.59*E* − 07	0.749
MAP2K6	333	216	227	−1.5	−1.5	−1.1	8.81*E* − 07	6.11*E* − 06	0.402
MAP3K5	676	470	479	−1.4	−1.4	−1.0	2.25*E* − 06	2.57*E* − 05	0.586
PPP1R12B	156	75	87	−2.1	−1.8	−1.2	4.35*E* − 06	2.71*E* − 05	0.125
MYC	1098	601	641	−1.8	−1.7	−1.1	4.76*E* − 06	1.63*E* − 04	0.494
PLCB1	382	216	239	−1.8	−1.6	−1.1	1.33*E* − 05	1.10*E* − 04	0.311
MAPKAPK5	963	654	644	−1.5	−1.5	1.0	2.36*E* − 05	1.59*E* − 05	0.708
ROCK2	681	501	506	−1.4	−1.4	−1.0	4.82*E* − 05	5.35*E* − 05	0.921
HDAC4	372	272	284	−1.4	−1.3	−1.0	1.12*E* − 04	4.58*E* − 04	0.476
